# Single or tandem autologous stem cell transplantation for treating Chinese patients with refractory/relapsed classical Hodgkin lymphoma

**DOI:** 10.1002/cam4.5765

**Published:** 2023-04-20

**Authors:** Chen Zhang, Jili Deng, Yan Xie, Lan Mi, Weiping Liu, Xiaopei Wang, Linjun Zhao, Yuqin Song, Jun Zhu

**Affiliations:** ^1^ Key Laboratory of Carcinogenesis and Translational Research (Ministry of Education/Beijing), Department of Lymphoma Peking University Cancer Hospital & Institute Beijing China; ^2^ Department of Lymphoma Peking University International Hospital Beijing China

**Keywords:** refractory or relapsed classical Hodgkin lymphoma, single autologous stem cell transplantation, tandem autologous stem cell transplantation, unfavorable risk

## Abstract

**Background:**

Autologous stem cell transplantation (ASCT) is the standard treatment strategy for refractory or relapsed classical Hodgkin lymphoma (R/R cHL). However, a single transplantation is insufficient to cure the disease because of unfavorable risk factors. Herein, we evaluated the outcomes of single or tandem ASCT in patients with R/R cHL, especially in high‐risk patients.

**Methods:**

We retrospectively analyzed R/R cHL patients who underwent single or tandem ASCT between April 2000 and June 2021 at the Beijing Cancer Hospital and Peking University International Hospital.

**Results:**

A total of 134 patients were enrolled. Patients were allocated to a favorable‐risk group (group A, *n* = 33), an unfavorable‐risk group (group B, *n* = 81) that underwent single ASCT, and an unfavorable‐risk group that underwent tandem ASCT (group C, *n* = 20). The median follow‐up time was 99 months (range, 91–107 months), and no treatment‐related deaths occurred after single or tandem ASCT. However, 27 patients (2 in group C) died during the follow‐up period. The groups A, B, and C had 5‐year progression‐free survival (PFS) rates of 77.05%, 45%, and 74.67%, respectively (*p* = 0.0014), and 5‐year overall survival (OS) rates of 89.85%, 76.06%, and 95%, respectively (*p* = 0.18). Neither the median PFS rates of groups A and C nor the OS rates of all groups were reached.

**Conclusions:**

Our study discusses the advantages of tandem transplantation for high‐risk patients with R/R cHL.

## Graphical abstract

Tandem ASCT improved the survival of unfavorable‐risk R/R cHL patients in China in the era of new drugs, and the efficacy evaluation status pre‐ASCT are critically important for identifying prognosis in patients with R/R cHL.

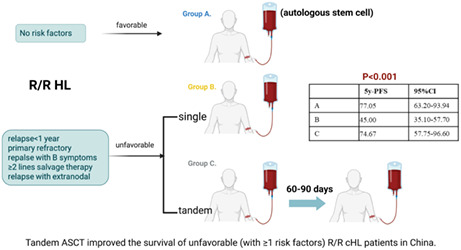

## 1 INTRODUCTION

Novel molecular therapeutic agents, such as brentuximab vedotin (BV), and immune checkpoint inhibitor, programmed death‐1 (PD‐1), such as nivolumab, enhance the prognosis of refractory or relapsed classical Hodgkin lymphoma (R/R cHL) and prolong survival after the failure of autologous stem cell transplantation (ASCT).^1^, ^2^, ^3^, ^4^, ^5^, ^6^, ^7^ High‐dose chemotherapy (HDCT) along with ASCT is the standard treatment strategy for cHL patients who fail first‐line therapies or have a relapse. Approximately half of patients with chemotherapy‐sensitive disease are cured with ASCT,^8^ whereas the other half, especially those with unfavorable risk factors, ultimately die because of disease progression and complications without successful treatment.^9^ The prognosis of patients with recurrence after ASCT is poor.^10^, ^11^, ^12^, ^13^ According to data from the European Society for Blood and Marrow Transplantation, the 5‐year overall survival (OS) is only 32%. Unfavorable‐risk factors include primary induction failure, extranodal involvement, B symptoms at relapse, disease relapse within 1 year of induction therapy, resistance to salvage therapy, and more than one salvage therapy regimen in patients with R/R cHL.^14^, ^15^, ^16^ However, a single transplantation is insufficient for long‐term control or to cure the disease because of unfavorable risk factors. Reducing recurrence after ASCT in high‐risk patients remains an issue in current research.

The AETHERA study found that consolidation with BV after ASCT improved the 5‐year progression‐free survival (PFS) of R/R cHL patients with unfavorable risk factors in comparison to the placebo.^14^, ^15^ BV consolidation treatment after transplantation has been used as a guideline in high‐risk patients. However, in China, few patients receive BV consolidation treatment because of its high cost. Checkpoint inhibitors are also important drugs in patients with R/R cHL. To date, only one small sample study has been reported; PD‐1 blockade administered as consolidation therapy after ASCT requires more data to confirm its effectiveness and safety.^17^

In addition to this new drug consolidation treatment, tandem transplantation was considered. However, the appropriate patients and opportunity for a second ASCT are unclear, since findings from previously published literature are limited.^18^, ^19^, ^20^, ^21^

Herein, we conducted a retrospective analysis to evaluate the outcomes of R/R cHL patients with at least one unfavorable prognostic factor after a single or tandem ASCT, and the significance of tandem transplantation in high‐risk patients. Furthermore, we compared the survival of favorable‐risk patients who received a single transplant in the same period.

## 2 PATIENTS AND METHODS

### 2.1 Patients

A total of 134 patients with R/R cHL with or without unfavorable prognostic factors, who underwent single or tandem ASCT between April 2000 and June 2021 at the Peking University Cancer Hospital & Institute and the Peking University International Hospital, were included in the analysis. Pathological diagnosis was confirmed in all patients by examination of biopsy materials according to the criteria of the WHO Classification of Hematological Malignancies. Data from the follow‐up period were retrospectively analyzed. This study was conducted in compliance with the Declaration of Helsinki and was approved by the Ethics Committee of the Peking University Cancer Hospital and the participating center. Written informed consent for participation was not required for this study, in accordance with national legislation and institutional requirements.

Unfavorable risks included cancer relapse within 1 year of induction therapy, primary induction failure, B symptoms at relapse, resistance to salvage therapy, more than one salvage therapy regimen before transplant, and extranodal involvement at relapse.^14^, ^15^, ^16^ Patients without (*n* = 33) and with (*n* = 81) at least one unfavorable risk factor underwent a single ASCT, whereas the remaining patients (*n* = 20) with at least one unfavorable risk factor underwent tandem ASCT. The decision on the type of transplant was made by the researchers. The number of stem cells collected was recorded. The baseline characteristics of the patients in groups B and C were similar. Patients in the single and tandem ASCT groups were infused with 1.75 × 10^6^ and 4.57 × 10^6^ CD34^+^ cells/kg (median values) of autologous peripheral blood progenitor cells, respectively. The preparatory regimen, stem cell apheresis procedures, and cryopreservation of stem cells were conducted in accordance with the institutional practice guidelines, and the data were retrospectively analyzed.

### 2.2 Treatment

All patients underwent a conditioning regimen followed by intravenous infusion of peripheral blood stem cells on day 0, and administration of 5 μg/kg of granulocyte colony‐stimulating factor (G–CSF) once a day from day +1 until the absolute neutrophil count (ANC) reached 2 × 10^9^/L. The first HDCT comprised the BEAM (carmustine 300 mg/m^2^/day, day −7; etoposide 100 mg/m^2^/q12h, day −6 to −3; cytarabine 100 mg/m^2^/q12h, day −6 to −3; melphalan 140 mg/m^2^/day, day −2), CBV (cyclophosphamide 1250 mg/m^2^/day, day −5 to −2; carmustine 300 mg/m^2^/day, day −6; etoposide 200 mg/m^2^/day, day −5 to −2), and BEAC (carmustine 300 mg/m^2^/day, day −6; etoposide 100 mg/m^2^/q12h, day −5 to −2; cytarabine 100 mg/m^2^/q12h, day −5 to −2; cyclophosphamide 1000 mg/m^2^/day, day −5 to −2). CBV therapy was the second most common conditioning regimen. In the tandem ASCT group (*n* = 20), the first conditioning regimen included BEAM (*n* = 14), CBV (*n* = 4), and BEAC (*n* = 2). A second conditioning regimen containing only CBV (*n* = 20) was maintained for patients without disease progression at that time. The interval between the two transplantation procedures was 60–90 days. Patients who underwent tandem transplantation successfully completed the procedure. No cancer recurrence occurred within 90 days in group B patients.

#### 2.2.1 Evaluation of efficacy and follow‐up

Responses were assessed before and after 6 weeks of ASCT according to the criteria of Lugano.^22^ Positron emission tomography and computed tomography (PET‐CT) scans were obtained and analyzed using the Deauville criteria.^23^ Follow‐up CT was performed every 3–6 months for 2 years and then semi‐annually or annually thereafter, and the scans were analyzed. OS was defined as the date of the first conditioning treatment regimen until death from any cause or the date of the last follow‐up. PFS was defined as the date of the first administration of the conditioning treatment regimen until cancer progression or the latest follow‐up date.

### 2.3 Statistical analysis

IBM SPSS Statistics software (Version 24.0, IBM Corp) and R version 4.1.2 (https://www.r‐project.org) were used for statistical analysis. The chi‐squared test or one‐way analysis of variance was performed to determine the significance of difference. PFS and OS rates were assessed using the Kaplan–Meier method and log‐rank tests. Univariate analysis was performed to analyze the baseline clinical characteristics and treatment programs to determine the significant association of prognostic variables with PFS and OS. Cox regression models were used to determine the significance of the influence of different prognostic variables identified using univariate and multivariate analyses. Statistical significance was set at *p* < 0.05.

## 3 RESULTS

### 3.1 Baseline and clinical characteristics of patients

The median age of the patients (*n* = 134) with R/R cHL who underwent single or tandem ASCT was 26 years (range, 20–34 years). The sex ratio was 1:1.8 (women:men). The most common pathological subtype was nodular sclerosis (71%), and 88 patients (66%) were initially at an advanced stage of cancer. Baseline characteristics such as stage, ECOG performance status, and B symptoms of all populations are matched; while group C patients are younger. Sixty‐three (47%) patients achieved complete response (CR), 51 (38%) achieved partial response (PR), and only 14 (10%) patients showed disease progression at the time of transplantation (Table 1). The baseline characteristics of patients in groups B and C, including age, sex, pathological subtype, stage, and ECOG performance status are matched. Analyze the high‐risk factors of groups B and C, the status at the time of recurrence, including extranodal involvement and B symptoms, is similar. There are also no statistical differences in the number of risk factors. Although a higher proportion of patients in group C were treated with intensive induction therapy such as BEACOPP, there were still more patients with primary drug resistance (25% vs. 75% *p* < 0.001) (Table 2). Since the approval of use of PD‐1 inhibitors in China in 2018 and that of BV in 2020, only eight patients have received these drugs as salvage therapy. Ninety‐six (71.6%) patients underwent PET‐CT, and 38 (28.4%) underwent CT examinations to evaluate their characteristics before ASCT. The median number of infused CD34^+^ cells was 2.01 cells/kg (range, 1.13–4.93 cells/kg). Stem cells were successfully transplanted into all the patients. The median time required for neutrophil (>0.5 × 10^9^/L) and platelet (>20 × 10^9^/L) recovery was 11 days (range, 10–12) and 11 days (range, 9–13), respectively.

**TABLE. 1:** Baseline characteristics of all populations

	*N*	Overall, *N* = 134	A (favorable, single ASCT) *N* = 33 (25%)^a^	B (unfavorable, single ASCT) *N* = 81 (60%)^a^	C (unfavorable, tandem ASCT) *N* = 20 (15%)^a^	*p*‐value^b^
Age	134	26 (20, 34)	32 (23, 36)	27 (20, 34)	24 (19, 26)	0.027
Sex	134					0.276
Female		48 (36%)	13 (39%)	31 (38%)	4 (20%)	
Male		86 (64%)	20 (61%)	50 (62%)	16 (80%)	
Pathological subtype	130					0.76
Lymphocyte‐rich		6 (4.6%)	2 (6.1%)	4 (5.1%)	0 (0%)	
Mixed cellularity		32 (25%)	9 (27%)	20 (26%)	3 (16%)	
Nodular sclerosis		92 (71%)	22 (67%)	54 (69%)	16 (84%)	
Stage	134					0.56
I		2 (1.5%)	1 (3.0%)	1 (1.2%)	0 (0%)	
II		44 (33%)	9 (27%)	31 (38%)	4 (20%)	
III		44 (33%)	12 (36%)	23 (28%)	9 (45%)	
IV		44 (33%)	11 (33%)	26 (32%)	7 (35%)	
Initial ECOG status	103					0.417
≦2		100 (97%)	26 (100%)	54 (95%)	20 (100%)	
>2		3 (2.9%)	0 (0%)	3 (5.3%)	0 (0%)	
Initial with B symptoms	129					0.832
No		66 (51%)	16 (52%)	41 (53%)	9 (45%)	
Yes		63 (49%)	15 (48%)	37 (47%)	11 (55%)	
Initial ESR elevated	68					0.261
No		22 (32%)	3 (19%)	10 (31%)	9 (45%)	
Yes		46 (68%)	13 (81%)	22 (69%)	11 (55%)	
Initial leukocyte count	77					0.43
No		37 (48%)	9 (47%)	16 (42%)	12 (60%)	
Yes		40 (52%)	10 (53%)	22 (58%)	8 (40%)	
Induce chemotherapy	134					0.125
ABVD		101 (75%)	24 (73%)	63 (78%)	14 (70%)	
BEACOPP		15 (11%)	5 (15%)	5 (6.2%)	5 (25%)	
Others		18 (13%)	4 (12%)	13 (16%)	1 (5.0%)	
Number of first‐line treatment cycles	133	6.00 (6.00, 7.00)	6.00 (6.00, 8.00)	6.00 (5.00, 7.00)	6.00 (4.75, 6.50)	0.202
Combined radiotherapy	134					0.548
No		88 (66%)	22 (67%)	55 (68%)	11 (55%)	
Yes		46 (34%)	11 (33%)	26 (32%)	9 (45%)	
Efficacy before ASCT	134					0.043
CR		63 (47%)	17 (52%)	38 (47%)	8 (40%)	
PR		51 (38%)	15 (45%)	31 (38%)	5 (25%)	
Less than PR		20 (15%)	1 (3.0%)	12 (15%)	7 (35%)	

^*a*^ Median (IQR); *n* (%).

^*b*^ Kruskal–Wallis rank sum test; Pearson's chi‐squared test; Fisher's exact test.

**TABLE. 2:** Baseline characteristics of unfavorable population (B & C).

	*N*	Overall, *N* = 101	B (unfavorable, single ASCT) *N* = 81 (80%)^a^	C (unfavorable, tandem ASCT) *N* = 20 (20%)^a^	*p*‐value^b^
Age	101	25 (20, 33)	27 (20, 34)	24 (19, 26)	0.058
Sex	101				0.124
Female		35 (35%)	31 (38%)	4 (20%)	
Male		66 (65%)	50 (62%)	16 (80%)	
Pathological subtype	97				0.484
Lymphocyte‐rich		4 (4.1%)	4 (5.1%)	0 (0%)	
Mixed cellularity		23 (24%)	20 (26%)	3 (16%)	
Nodular sclerosis		70 (72%)	54 (69%)	16 (84%)	
Stage	101				0.36
I		1 (1.0%)	1 (1.2%)	0 (0%)	
II		35 (35%)	31 (38%)	4 (20%)	
III		32 (32%)	23 (28%)	9 (45%)	
IV		33 (33%)	26 (32%)	7 (35%)	
Initial ECOG status	77				0.564
≦2		74 (96%)	54 (95%)	20 (100%)	
>2		3 (3.9%)	3 (5.3%)	0 (0%)	
Initial with B symptoms	98				0.546
No		50 (51%)	41 (53%)	9 (45%)	
Yes		48 (49%)	37 (47%)	11 (55%)	
Initial ESR elevated	52				0.382
No		19 (37%)	10 (31%)	9 (45%)	
Yes		33 (63%)	22 (69%)	11 (55%)	
Initial leukocyte count	58				0.195
No		28 (48%)	16 (42%)	12 (60%)	
Yes		30 (52%)	22 (58%)	8 (40%)	
Induce chemotherapy	101				0.038
ABVD		77 (76%)	63 (78%)	14 (70%)	
BEACOPP		10 (9.9%)	5 (6.2%)	5 (25%)	
Others		14 (14%)	13 (16%)	1 (5.0%)	
Number of first‐line treatment cycles	101	6.00 (5.00, 7.00)	6.00 (5.00, 7.00)	6.00(4.75, 6.50)	0.964
Combined radiotherapy	101				0.278
No		66 (65%)	55 (68%)	11 (55%)	
Yes		35 (35%)	26 (32%)	9 (45%)	
Efficacy before ASCT	101				>0.999
CR		46 (45%)	38 (47%)	8 (40%)	
PR		36 (36%)	31 (38%)	5 (25%)	
Less than PR		19 (19%)	12 (15%)	7 (35%)	
High‐risk factors					
Refractory	101				<0.001
No		66 (65%)	61 (75%)	5 (25%)	
Yes		35(35%)	20 (25%)	15 (75%)	
Relapse within 12 months	101				<0.001
No		51 (50%)	33 (41%)	18 (90%)	
Yes		50 (50%)	48 (59%)	2 (10%)	
Relapse with B symptoms	101				0.74
No		84 (83%)	68 (84%)	16 (80%)	
Yes		17 (17%)	13 (16%)	4 (20%)	
Relapse with extranodal involvement	101				>0.999
No		89 (88%)	71 (88%)	18 (90%)	
Yes		12(12%)	10 (12%)	2 (10%)	
More than one salvage therapy regimen before transplantation	101				0.21
No		69 (68%)	53 (65%)	16 (80%)	
Yes		32 (32%)	28 (35%)	4 (20%)	
Number of risk factors	101				0.311
1		66(65%)	51(63%)	15(75%)	
>1		35(35%)	30(37%)	5(25%)	

^*a*^ Median (IQR); *n* (%).

^*b*^ Kruskal–Wallis rank sum test; Pearson's chi‐squared test; Fisher's exact test.

### 3.2 Responses and survival

Seventy‐six (66.7%) patients achieved CR, and 46 (22.8%) achieved PR after one course of ASCT (groups A and B). In group C, 40% of patients achieved CR before the onset of ASCT, and after the first and second transplants, their CR rates increased to 75% and 90%, respectively. Ninety‐six and 38 patients underwent PET‐CT scanning 6 weeks and CT scans 4 weeks after ASCT, respectively. In addition, 93 (69%) patients in both groups achieved CR.

**FIGURE 1:**
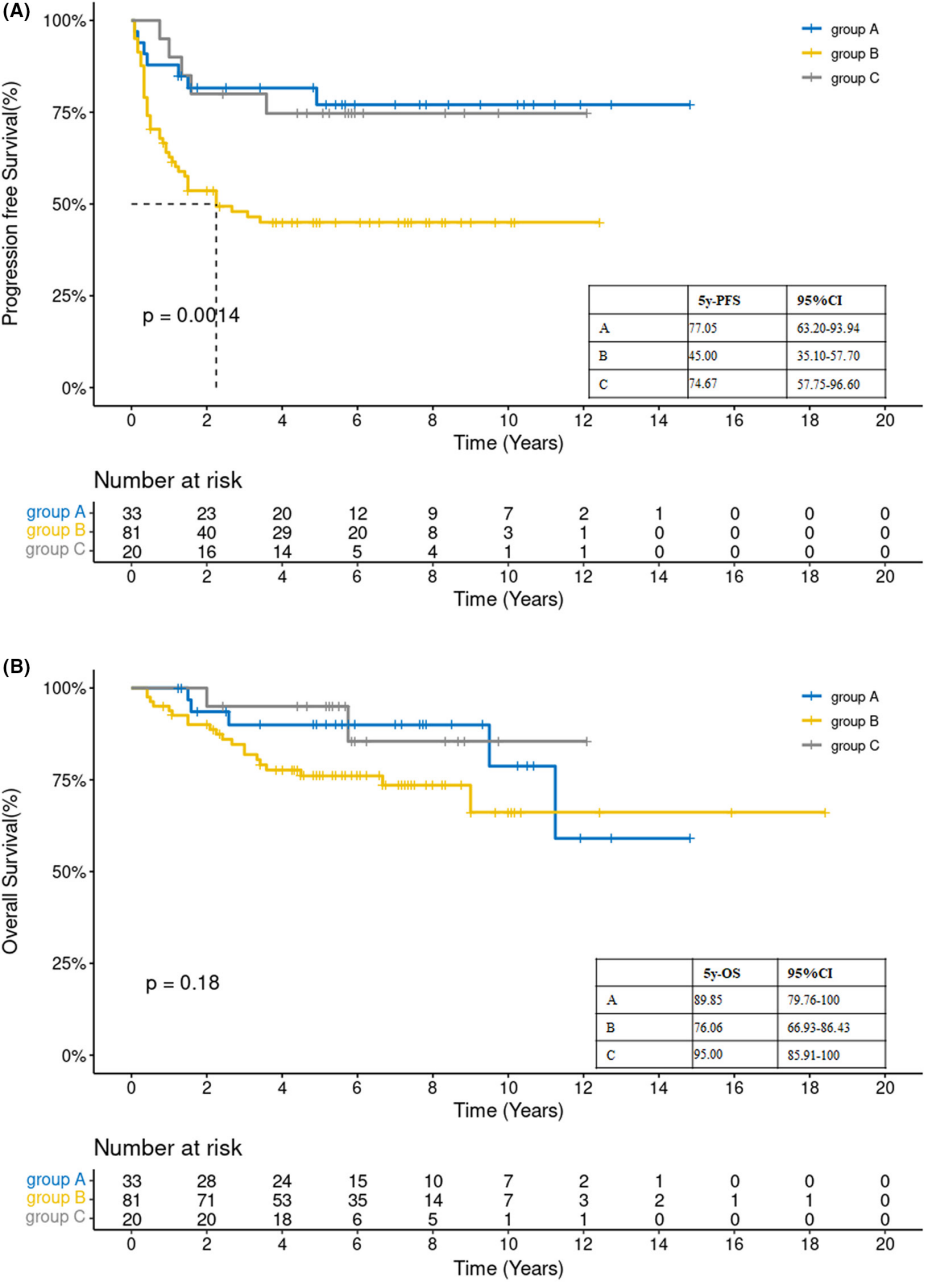
The 5‐year (a) progression‐free survival (PFS) and (b) overall survival (OS) rates of the three groups. Group A—without unfavorable risk and single autologous stem cell transplantation (ASCT). Group B—with unfavorable risk and single ASCT. Group C—with unfavorable risk and tandem ASCT. CR, complete response; PR, partial response.

**FIGURE 2:**
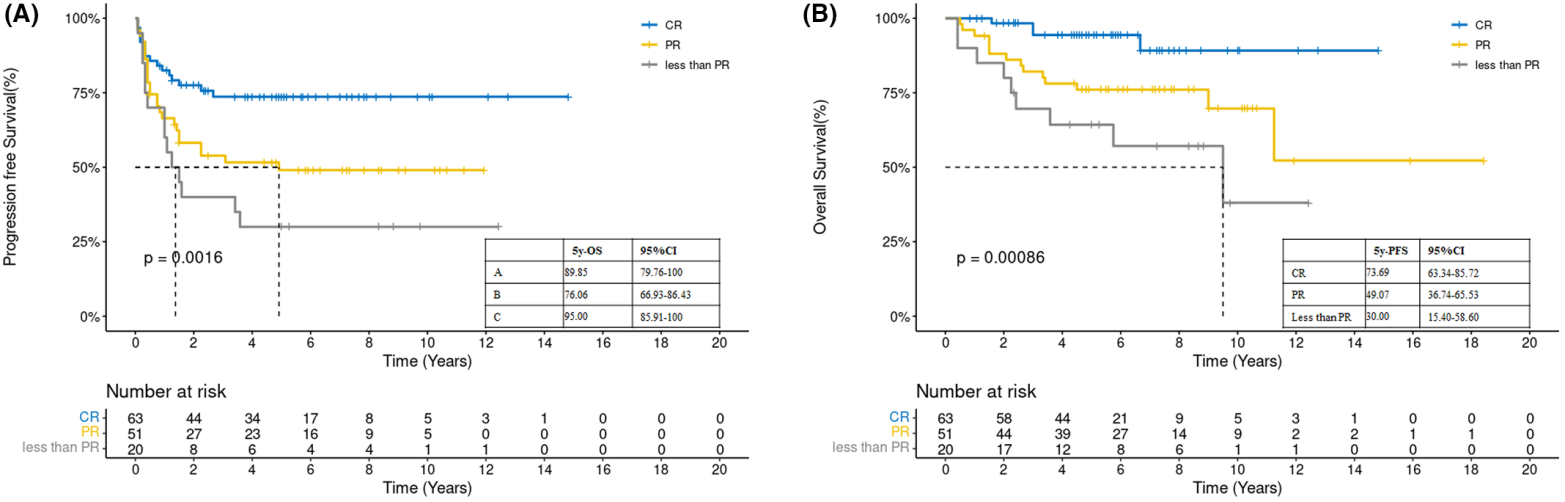
The 5‐year (a) progression‐free survival (PFS) and (b) overall survival (OS) rates of patients who achieved a complete response (CR) or partial response (PR) or less than PR status before autologous stem cell transplantation.

During the follow‐up period (median, 99 months; range, 91–107), 27 patients (2 in group C) died, including 25 deaths caused by lymphoma and 2 caused by pneumonia (in group B). Among the patients (*n* = 134) included in the survival analysis, the 5‐year OS and PFS rates were 82.4% and 57.3%, respectively. A significant difference in PFS (*p* = 0.0246), but not OS, was found between groups B and C. The 2‐year PFS rates in groups B and C were 53.6% and 80%, respectively. The 5‐year PFS rates of groups A, B, and C were 77.05%, 45%, and 74.67%, respectively (*p* = 0.0014), and their 5‐year OS rates were 89.85%, 76.06%, and 95%, respectively (*p* = 0.18) (Figure 1). Neither the median PFS of groups A and C nor the median OS of all three groups were reached. The 5‐year PFS rates of patients in the three groups who experienced CR, PR, or less than PR before ASCT were 73.69%, 49.07%, and 30%, respectively (*p* = 0.0016). Their 5‐year OS rates were 94.4%, 76.04%, and 64.3%, respectively (*p* = 0.00086) (Figure 2).

In group A, seven patients relapsed and two patients were treated with PD‐1 inhibitors. Among the 43 patients with recurrence in group B, 16 patients were given PD‐1 inhibitors, two patients were given BV, and two patients were given both two. While in group C, one recurrent patient in five received BV and the other four patients received PD‐1 inhibitors (most of them participated in clinical trials). In addition, there were two patients in group B who received allogeneic transplantation.

**FIGURE 3:**
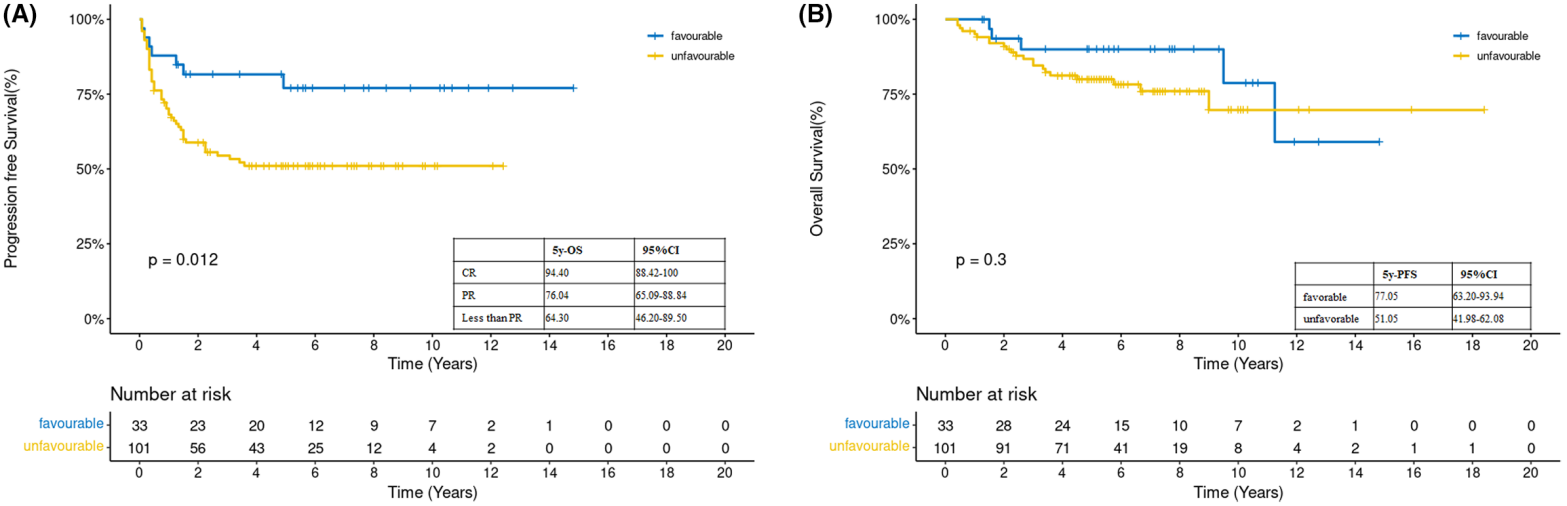
(a) Progression‐free survival (PFS) and (b) overall survival (OS) at 5 years in patients with or without unfavorable risks.

Pre‐transplant data of the PET‐CT scans of 96 patients were available for analysis. The pre‐transplant 5‐year OS and PFS rates of the PET‐CT‐negative patients were 95.5% and 72.32%, respectively, whereas those of the PET‐CT‐positive patients were 76.1% and 51.7%, respectively (OS, *p* = 0.0061; PFS, *p* = 0.0063). Ninety‐six patients underwent post‐transplant PET‐CT. The 5‐year OS and PFS rates of post‐transplant PET‐CT‐negative patients were 95.46% and 76.95%, respectively, whereas those of the PET‐CT‐positive patients were 59.3% and 19.48%, respectively (OS, *p* < 0.0001; PFS, *p* < 0.0001). The 5‐year PFS of patients with or without unfavorable risk factors was 51.05% and 77.05%, respectively (*p* = 0.012), and the 5‐year OS rates were 89.95% and 80%, respectively (*p* = 0.3) (Figure 3). The number of previous chemotherapy regimens affected PFS, but not OS. Thus, the 5‐year PFS rates of patients who underwent more than or less than two regimen lines were 34.4% and 64.62%, respectively (*p* = 0.00055).

### 3.3 Identification of prognostic factors

Factors that were significantly associated with PFS and OS after ASCT with a univariate model were analyzed using a multivariate model. Univariate analysis revealed that patients with unfavorable factors (*p* = 0.017; hazard ratio [HR] = 2.64) in group B (*p* = 0.023; HR = 2.94) achieved CR or PR before transplantation (*p* = 0.008; HR = 2.29). Initial Eastern Cooperative Oncology Group (ECOG) status (*p* = 0.003; HR = 6.09), B symptoms at the time of diagnosis (*p* = 0.021; HR = 1.91), number of previous chemotherapy regimen lines before transplantation (*p* = 0.001; HR = 2.94), and PET‐CT status post‐ASCT significantly influenced PFS. Achieving CR or PR after transplantation (*p* = 0.005; HR = 3.16), PET‐CT status pre‐ASCT (*p* = 0.017; HR = 6.32), and post‐ASCT (*p* < 0.0001; HR = 11.06) significantly influenced OS (Table 3). Additionally, univariate analysis showed no significant difference in the number of transplants; however, a trend of survival benefit was identified. Multivariate analysis revealed that group B (*p* = 0.014; HR = 4.78), initial ECOG status >1 (*p* = 0.004; HR = 11.75), and PET‐CT positive status post‐ASCT (*p* = 0.009; HR = 3.42) significantly and adversely influenced PFS. Post‐transplantation PET‐CT‐positive status (*p* = 0.006; HR = 7.39) was significantly associated with an adverse influence on OS (Table 4).

**TABLE 3:** Factors significantly associated with OS and PFS after ASCT in univariate analysis

		Univariate					
		PFS			OS		
	Characteristics	HR	95% CI	*p*‐value	HR	95% CI	*p*‐value
1	With unfavorable factors	2.64	1.19–5.84	0.017	1.68	0.63–4.48	0.301
2	Group A vs. C^a^	0.92	0.29–2.91	0.894	1.42	0.27–7.38	0.675
3	Group B vs. C^a^	2.94	1.16–7.44	0.023	2.77	0.65–11.85	0.17
4	Initial ECOG status>1	6.09	1.84–20.2	0.003	–		
5	Initial with B symptom	1.91	1.1–3.3	0.021	–		
6	Initial leukocyte count	2.08	0.96–4.5	0.065	–		
7	Salvage therapy regimen before transplantation>1^b^	2.53	1.47–4.38	0.001	1.79	0.79–4.05	0.162
8	CR + PR vs. <PR before transplantation	2.29	1.25–4.2	0.008	3.16	1.42–7.03	0.005
9	PET‐CT pre‐ASCT	1.9	0.96–3.77	0.065	6.32	1.38–28.83	0.017
10	PET‐CT post‐ASCT	6.28	3.17–12.46	<0.0001	11.06	2.98–40.96	<0.0001
11	Single ASCT vs. tandem ASCT	0.45	0.18–1.12	0.085	0.43	0.1–1.81	0.248

^a^ Group A‐without unfavorable‐risk and single ASCT, Group B‐with unfavorable‐risk and single ASCT, Group C‐with unfavorable‐risk and tandem ASCT

^b^ Number of lines for salvage therapy regimen

**TABLE 4:** Factors significantly associated with OS and PFS after ASCT in multivariate analysis

		**Multivariate**					
		**PFS**			**OS**		
	Characteristics	HR	CI 95%	*p*‐value	HR	CI 95%	*p*‐value
1	With unfavorable factors	0.32	0.05‐1.91	0.21	‐		
2	Group A *vs*. C^a^	‐			‐		
3	**Group B *vs*. C** ^a^	**4.78**	**1.37‐16.7**	**0.014**	‐		
4	CR+PR *vs*. <PR before transplantation	2.99	1.07‐8.34	0.036	1.74	0.5‐6.07	0.386
5	**initial ECOG status>1**	**11.75**	**2.22‐62.06**	**0.004**	‐		
6	Initial with B symptom	1.25	0.55‐2.83	0.59	‐		
7	Number of previous chemotherapy regimen lines before transplantation>2	2.01	0.82‐4.94	0.127	‐		
8	PET‐CT pre‐ASCT	‐			2.21	0.39‐12.59	0.373
9	**PET‐CT post‐ASCT**	**3.42**	**1.36‐8.69**	**0.009**	**7.39**	**1.77‐30.89**	**0.006**

^a^ Group A—without unfavorable‐risk and single ASCT; group B—with unfavorable‐risk and single ASCT; group C—with unfavorable‐risk and tandem ASCT.

## 4 DISCUSSION

cHL is highly treatable with initial chemotherapy. However, 10%–35% of patients experience disease progression.^24^, ^25^ Treatment of patients with R/R cHL remains a challenge despite the use of novel molecular therapies, even after BV and immune checkpoint inhibitors have been approved for the treatment of R/R patients undergoing ASCT. However, the role of ASCT in the treatment of R/R cHL has been well‐established. Approximately half of patients with chemotherapy‐sensitive disease are cured with ASCT,^8^ whereas the other half, especially those with unfavorable risk factors, exhibit disease progression and have poor outcomes.^9^ The following unfavorable factors are defined as high‐risk: primary induction failure, extranodal involvement, B symptoms at relapse, disease relapse within 1 year of induction therapy, resistance to salvage therapy, and more than one salvage therapy regimen before transplantation.^14^, ^15^, ^16^ Attempts are being made to improve the survival of high‐risk patients through new drug consolidation or sequential transplantation. Our study discusses the advantages of tandem transplantation for high‐risk patients with R/R cHL. This kind of treatment is not only effective but also accessible and affordable.

The AETHERA study found that early consolidation with BV after ASCT improved the 5‐year PFS of R/R cHL patients with unfavorable risk factors in comparison to placebo (59% vs. 41%, respectively; HR 0.521).^14^, ^15^ BV consolidation treatment after transplantation has been used as a guideline in high‐risk patients. However, in China, few patients receive the treatment because of its high cost. Checkpoint inhibitors are also important drugs for treating patients with R/R cHL after ASCT. Two recently published related phase II trials, CheckMate 205 and KEYNOTE‐087, have demonstrated median PFS rates of 14.7 and 13.7 months, respectively.^3^, ^4^ However, the efficacy duration of the checkpoint inhibitors remains far from satisfactory. In a phase II study with a small sample size,^17^ 30 patients received PD‐1 blockade treatment after ASCT for eight cycles. Overall improvement in PFS at 18 months after ASCT from the estimated historical results was in the range of 60%–82%; therefore, the author concluded that checkpoint inhibitor consolidation is a successful method for R/R cHL patients post‐ASCT. This phase II study has some limitations. First, the sample size was small, and this was not a randomized controlled study. Second, not all the patients belonged to high‐risk groups, and 87% of them met the eligibility criteria for the high‐risk AETHERA study. Thus, the follow‐up time was short. PD‐1 blockade administration consolidation therapy after ASCT requires more data to confirm its effectiveness and safety.

In addition to the treatment involving consolidation with new drugs, tandem transplantation was also considered as an important regimen. Limited data are available regarding the use of tandem therapy in unfavorable R/R cHL patients; however, the H96 trial by the LYSA/SFGM‐TC study group had a large sample size and long follow‐up duration.^19^ This multicenter phase II trial included 245 primary refractory or first‐relapse cHL patients eligible for single or tandem ASCT, and confirmed the feasibility of tandem transplantation for high‐risk patients. Nevertheless, the H96 trial had some limitations. PET was not included in the assessment criteria in the beginning of the trial, and only patients with first‐line treatment failure were included. Patients with resistance to salvage therapy or persistent disease before transplantation were not included. The 5‐year PFS rate was 95% in our study, whereas the 10‐year PFS rate was 47% in the H96 trial. However, direct comparisons cannot be drawn between the two studies because the baseline information and follow‐up duration were inconsistent. The results of this study demonstrated that R/R cHL patients with an unfavorable prognosis achieved long‐term remission or cure after undergoing tandem ASCT, with favorable treatment outcomes. Forty‐six R/R cHL patients with high‐risk factors were included in another study, of which 41 received tandem ASCT and reported a 5‐year PFS of 49%,^20^ while we observed a 74.67% 5‐year PFS rate in our study. The following differences were noted between the two studies: the duration when the study was conducted (our study being more recent); the definition of high‐risk patients (our inclusion criteria comprised more high‐risk factors and were widely acceptable); and the survival rate of the patients (which showed greater improvement in our study). However, the sample size of both the studies was relatively small. Our results were also superior to those of the SWOG 0410 trial, in which 82 patients with R/R cHL received tandem transplantation (5‐year PFS, 55%).^21^ These differences may be due to variability in the inclusion criteria of patients and sample size.

Previous studies have also discussed the conditioning regimen for transplantation of R/R cHL patients. The choice of conditioning regimen for ASCT II has traditionally been based on institutional experience. Several regimens such as BEAM, CBV, BuCy, and TBI‐containing are considered standard and routinely used for lymphoma patients. A large study from CIBMTR showed^26^ that patients with HL receiving BEAM had superior overall survival compared to all other regimens, probabilities of 3y‐OS were: BEAM 79%, CBV 68%, BuCy 65%, and TBI 47% (*p* < 0.001). In our study, preference will be given to BEAM for ASCT I, other classical conditioning regimens are an option for ASCT II, CBV in particular.

In the present study, the 5‐year PFS of unfavorable‐risk patients who received a single ASCT was 45%, similar to that of the placebo group in the AETHERA study.^14^, ^15^ Moreover, we observed a 5‐year PFS rate of 75% in patients with unfavorable risk factors who were treated with tandem ASCT, which was higher than that in patients treated with BV consolidation. On comparison, tandem ASCT was superior to BV consolidation after a single course of ASCT in patients with unfavorable risk factors. There is a report of anti‐PD‐1 consolidation on small sample size recently, with a 2‐year PFS and OS rates of 79% and 87%, respectively.^27^ Compare with our study, the 2‐year PFS and OS rates were 80% (95% CI 64–100) and 95% (95% CI 86–100), respectively. There is also report of the two drugs combination, BV combined with anti‐PD‐1 was given for consolidation of high‐risk patients after transplantation. At a median follow‐up of 29.9 months, the 18‐month PFS was 94% (95% CI 84–98).^28^ In China, the cost of BV consolidation treatment is at least five times that of ASCT, and tandem transplantation has considerable practical significance for unfavorable‐risk R/R cHL patients. Nevertheless, the present study was not a randomized study that compared tandem ASCT with BV consolidation. Furthermore, the baseline characteristics of the populations in different studies were not similar. Our study was retrospective and did not directly compare various treatment regimens. Additionally, the sample size was small. Therefore, whether tandem ASCT can replace BV consolidation in these patients remains unclear. However, tandem transplantation costs less and has higher acceptance in China. In future, we plan to conduct a randomized controlled study with a larger sample size to confirm the practical significance of tandem ASCT.

Nevertheless, tandem ASCT is a viable option for treating patients with unfavorable prognosis in the present era of new molecular therapeutic agents. Treatment strategies such as consolidation with new drugs, tandem‐autoSCT or auto‐alloSCT,^29^, ^30^, ^31^ and radiation therapy^32^ can improve patient outcomes after ASCT. The clinical trials designed by us in the future will randomly group unfavorable patients who have already undergone ASCT1 to receive ASCT2, BV, immune checkpoint inhibitors, radiation, or allogeneic stem cell transplantation.

The results of the current trial demonstrate a risk‐adapted strategy for patients with R/R cHL. In our study, it was confirmed that tandem ASCT improved the PFS of unfavorable‐risk patients compared with single transplantation, but there was no significant difference in overall survival. A higher proportion of patients in single ASCT group were subsequently treated with BV and PD1‐blockers (46.5% vs. 40%), In addition, there were two patients in single ASCT group who received allogeneic transplantation. This may be the reason for the lack of OS differences across groups in our study. In addition, our study focused on the prognostic significance of PET/CT results. Pre‐ASCT PET‐CT findings are critically important for identifying prognosis in patients with R/R cHL.^33^, ^34^, ^35^, ^36^, ^37^ For example, a previous study revealed that the findings from PET scan images were associated with PFS in patients with R/R cHL, which is consistent with the data from our study. Here, the 5‐year PFS rates of patients who achieved CR or PR before ASCT were 73.69% and 49.07%, respectively, although the 5‐year PFS rates of patients with SD and PD were 30%. Therefore, patients may achieve a PR or better response before undergoing ASCT.

## 5 CONCLUSION

In the era of new drugs, the aim of this multicenter retrospective study with a long follow‐up duration was to improve the survival of unfavorable‐risk R/R cHL patients in China by means of tandem ASCT and compare the data with those reported in previous studies to confirm its effectiveness and safety. The favorable outcomes of tandem ASCT make it a treatment option for unfavorable‐risk R/R cHL patients.

## AUTHOR CONTRIBUTIONS

**Chen Zhang:** Conceptualization (equal); data curation (lead); formal analysis (equal); investigation (lead); methodology (equal); project administration (lead); resources (lead); software (equal); supervision (equal); validation (equal); visualization (equal); writing – original draft (lead); writing – review and editing (lead). **Jili Deng:** Data curation (supporting); formal analysis (equal); methodology (supporting); software (supporting); supervision (supporting); validation (supporting). **Yan Xie:** Data curation (supporting); investigation (supporting); methodology (supporting); resources (supporting). **Lan Mi:** Formal analysis (supporting); methodology (supporting); writing – review and editing (supporting). **Weiping Liu:** Conceptualization (supporting); writing – review and editing (supporting). **Xiaopei Wang:** Conceptualization (supporting); writing – review and editing (supporting). **Linjun Zhao:** Data curation (supporting); resources (supporting). **Yuqin Song:** Conceptualization (supporting). **Jun Zhu:** Conceptualization (equal); funding acquisition (lead); project administration (supporting); writing – review and editing (supporting).

## FUNDING INFORMATION

This study was supported by the National Natural Science Foundation of China (no. 82070205).

## CONFLICT OF INTEREST STATEMENT

The authors declare no conflict of interests.

## Data Availability

The datasets generated during and analysed during the current study are available from the corresponding author on reasonable request.

## References

[1] Chen R , Gopal AK , Smith SE , et al. Five‐year survival and durability results of brentuximab vedotin in patients with relapsed or refractory Hodgkin lymphoma. Blood. 2016;128(12):1562‐1566. 27432875 10.1182/blood-2016-02-699850 PMC5034737

[2] Chen R , Zinzani PL , Fanale MA , et al. Phase II study of the efficacy and safety of pembrolizumab for relapsed/refractory classic Hodgkin lymphoma. J Clin Oncol. 2017;35(19):2125‐2132. 28441111 10.1200/JCO.2016.72.1316 PMC5791843

[3] Armand P , Engert A , Younes A , et al. Nivolumab for relapsed/refractory classic Hodgkin lymphoma after failure of autologous hematopoietic cell transplantation: extended follow‐up of the multicohort single‐arm phase II CheckMate 205 trial. J Clin Oncol. 2018;36(14):1428‐1439. 29584546 10.1200/JCO.2017.76.0793 PMC6075855

[4] Chen R , Zinzani PL , Lee HJ , et al. Pembrolizumab in relapsed or refractory Hodgkin lymphoma: 2‐year follow‐up of KEYNOTE‐087. Blood. 2019;134(14):1144‐1153. 31409671 10.1182/blood.2019000324 PMC6776792

[5] Kuruvilla J , Ramchandren R , Santoro A , et al. Pembrolizumab versus brentuximab vedotin in relapsed or refractory classical Hodgkin lymphoma (KEYNOTE‐204): an interim analysis of a multicentre, randomised, open‐label, phase 3 study. Lancet Oncol. 2021;22(4):512‐524. 33721562 10.1016/S1470-2045(21)00005-X

[6] Advani RH , Moskowitz AJ , Bartlett NL , et al. Brentuximab vedotin in combination with nivolumab in relapsed or refractory Hodgkin lymphoma: 3‐year study results. Blood. 2021;138(6):427‐438. 33827139 10.1182/blood.2020009178 PMC12684812

[7] Diefenbach CS , Hong FX , Ambinder RF , et al. Ipilimumab, nivolumab, and brentuximab vedotin combination therapies in patients with relapsed or refractory Hodgkin lymphoma: phase 1 results of an open‐label, multicentre, phase 1/2 trial. Lancet Haematol. 2020;7(9):e660‐e670. 32853585 10.1016/S2352-3026(20)30221-0 PMC7737486

[8] Perales MA , Ceberio I , Armand P , et al. Role of cytotoxic therapy with hematopoietic cell transplantation in the treatment of Hodgkin lymphoma: guidelines from the American Society for Blood and Marrow Transplantation. Biol Blood Marrow Transplant. 2015;21(6):971‐983. 25773017 10.1016/j.bbmt.2015.02.022

[9] Rimner A , Lovie S , Hsu M , et al. Accelerated total lymphoid irradiation‐containing salvage regimen for patients with refractory and relapsed Hodgkin lymphoma: 20 years of experience. Int J Radiat Oncol Biol Phys. 2017;97(5):1066‐1076. 28332991 10.1016/j.ijrobp.2017.01.222 PMC5474094

[10] Di Renzo N , Gaudio F , Carlo Stella C , et al. Relapsing/refractory HL after auto transplantation: which treatment? Acta Biomed. 2020;91(S‐5):30‐40. 32525132 10.23750/abm.v91iS-5.9912 PMC7944654

[11] Sureda A , André M , Borchmann P , et al. Improving outcomes after autologous transplantation in relapsed/refractory Hodgkin lymphoma: a European expert perspective. BMC Cancer. 2020;20(1):1088‐1102. 33172440 10.1186/s12885-020-07561-2 PMC7657361

[12] Bentolila G , Pavlovsky A . Relapse or refractory Hodgkin lymphoma: determining risk of relapse or progression after autologous stem‐cell transplantation. Leuk Lymphoma. 2020;61(7):1548‐1554. 32148142 10.1080/10428194.2020.1732959

[13] Bento L , Boumendil A , Finel H , et al. Tandem autologous‐reduced intensity allogeneic stem cell transplantation in high‐risk relapsed Hodgkin lymphoma: a retrospective study of the Lymphoma Working Party‐EBMT. Bone Marrow Transplant. 2021;56(3):655‐663. 33046830 10.1038/s41409-020-01075-y

[14] Moskowitz CH , Nademanee A , Masszi T , et al. Brentuximab vedotin as consolidation therapy after autologous stem‐cell transplantation in patients with Hodgkin's lymphoma at risk of relapse or progression (AETHERA): a randomised, double‐blind, placebo‐controlled, phase 3 trial. Lancet. 2015;385:1853‐1862. 25796459 10.1016/S0140-6736(15)60165-9

[15] Moskowitz CH , Walewski J , Nademanee A , et al. Five‐year PFS from the AETHERA trial of brentuximab vedotin for Hodgkin lymphoma at high risk of progression or relapse. Blood. 2018;132(25):2639‐2642. 30266774 10.1182/blood-2018-07-861641

[16] Massano D , Carraro E , Mussolin L , et al. Brentuximab vedotin in the treatment of paediatric patients with relapsed or refractory Hodgkin's lymphoma: results of a real‐life study. Pediatr Blood Cancer. 2022 Jun;2:e29801. 10.1002/pbc.29801 35656841

[17] 17 Armand P , Chen YB , Redd RA , et al. PD‐1 blockade with pembrolizumab for classical Hodgkin lymphoma after autologous stem cell transplantation. Blood. 2019;134:22‐29. 30952672 10.1182/blood.2019000215 PMC6609955

[18] Smith SM , van Besien K , Carreras J , et al. Second autologous stem cell transplantation for relapsed lymphoma after a prior autologous transplant. Biol Blood Marrow Transplant. 2008;14(8):904‐912. 18640574 10.1016/j.bbmt.2008.05.021 PMC3353768

[19] Morschhauser F , Brice P , Ferme C , et al. Risk‐adapted salvage treatment with single or tandem autologous stem‐cell transplantation for first relapse/refractory Hodgkin's lymphoma: results of the prospective multicenter H96 trial by the GELA/SFGM study group. J Clin Oncol. 2008;26:5980‐5987. 19018090 10.1200/JCO.2007.15.5887

[20] Fung HC , Stiff P , Schriber J , et al. Tandem autologous stem cell transplantation for patients with primary refractory or poor risk recurrent Hodgkin lymphoma. Biol Blood Marrow Transplant. 2007;13:594‐600. 17448919 10.1016/j.bbmt.2007.01.072

[21] Smith EP , Li H , Friedberg JW , et al. Tandem autologous hematopoietic cell transplantation for patients with primary progressive or recurrent Hodgkin lymphoma: a SWOG and blood and marrow transplant clinical trials network phase II trial (SWOG S0410/BMT CTN 0703). Biol Blood Marrow Transplant. 2018;24(4):700‐707. 29289757 10.1016/j.bbmt.2017.12.798 PMC5965270

[22] Cheson BD , Fisher RI , Barrington SF , et al. Recommendations for initial evaluation, staging, and response assessment of Hodgkin and non‐Hodgkin lymphoma: the Lugano classification. J Clin Oncol. 2014;32(27):3059‐3068. 25113753 10.1200/JCO.2013.54.8800 PMC4979083

[23] Meignan M , Barrington S , Itti E , Gallamini A , Haioun C , Polliack A . Report on the 4th International Workshop on Positron Emission Tomography in Lymphoma held in Menton, France, 3‐5 October 2012. Leuk Lymphoma. 2014;55(1):31‐37. 23763460 10.3109/10428194.2013.802784

[24] Lim SH , Johnson PWM . Optimizing therapy in advanced‐stage Hodgkin lymphoma. Blood. 2018;131(15):1679‐1688. 29500173 10.1182/blood-2017-09-772640

[25] Bröckelmann PJ , Sasse S , Engert A . Balancing risk and benefit in early‐stage classical Hodgkin lymphoma. Blood. 2018;131(15):1666‐1678. 29500174 10.1182/blood-2017-10-772665

[26] Chen YB , Lane AA , Logan B , et al. Impact of conditioning regimen on outcomes for patients with lymphoma undergoing high‐dose therapy with autologous hematopoietic cell transplantation. Biol Blood Marrow Transplant. 2015;21(6):1046‐1053. 25687795 10.1016/j.bbmt.2015.02.005 PMC4426014

[27] De Filippi R , Marcacci G , Derenzini E , et al. Anti‐PD1 consolidation in patients with Hodgkin lymphoma at high risk of relapse after autologous stem cell transplantation: a multicenter real‐life study. Cancers (Basel). 2022;14(23):5846. 36497328 10.3390/cancers14235846 PMC9739754

[28] Herrera AF , Chen L , Nieto Y , et al. Brentuximab vedotin plus nivolumab after autologous haematopoietic stem‐cell transplantation for adult patients with high‐risk classic Hodgkin lymphoma: a multicentre, phase 2 trial. Lancet Haematol. 2023 Jan;10(1):e14‐e23. 36403579 10.1016/S2352-3026(22)00318-0

[29] Sureda A , Genadieva Stavrik S , Boumendil A , et al. Changes in patients population and characteristics of hematopoietic stem cell transplantation for relapsed/refractory Hodgkin lymphoma: an analysis of the lymphoma working party of the EBMT. Bone Marrow Transplant. 2020;55(11):2170‐2179. 32415225 10.1038/s41409-020-0929-y

[30] Badar T , Epperla N , Szabo A , et al. Trends in postrelapse survival in classic Hodgkin lymphoma patients after experiencing therapy failure following auto‐HCT. Blood Adv. 2020;4(1):47‐54. 31899797 10.1182/bloodadvances.2019000736 PMC6960457

[31] Ansell SM . Hodgkin lymphoma: a 2020 update on diagnosis, risk‐stratification, and management. Am J Hematol. 2020;95(8):978‐989. 32384177 10.1002/ajh.25856

[32] Constine LS , Yahalom J , Ng AK , et al. The role of radiation therapy in patients with relapsed or refractory Hodgkin lymphoma: guidelines from the International Lymphoma Radiation Oncology Group. Int J Radiat Oncol Biol Phys. 2018;100(5):1100‐1118. 29722655 10.1016/j.ijrobp.2018.01.011

[33] Adams HJ , Kwee TC . Prognostic value of pretransplant FDG‐PET in refractory/relapsed Hodgkin lymphoma treated with autologous stem cell transplantation: systematic review and meta‐analysis. Ann Hematol. 2016;95(5):695‐706. 26931115 10.1007/s00277-016-2619-9 PMC4819743

[34] Procházka V , Gawande RS , Cayci Z , et al. Positron emission tomography‐based assessment of metabolic tumor volume predicts survival after autologous hematopoietic cell transplantation for Hodgkin lymphoma. Biol Blood Marrow Transplant. 2018;24(1):64‐70. 28942016 10.1016/j.bbmt.2017.09.006 PMC6431258

[35] Mokrane FZ , Chen A , Schwartz LH , et al. Performance of CT compared with ^18^F‐FDG PET in predicting the efficacy of nivolumab in relapsed or refractory Hodgkin lymphoma. Radiology. 2020;295(3):651‐661. 32286191 10.1148/radiol.2020192056

[36] Texte E , Lequesne J , Tilly H , et al. SUVmax‐based assessment of PET response shows a superior specificity to Deauville criteria for predicting recurrence in Hodgkin's lymphoma. Leuk Lymphoma. 2021;62(5):1088‐1097. 33289431 10.1080/10428194.2020.1855341

[37] Kedmi M , Khaustov P , Ribakovsy E , Benjamini O , Avigdor A . Outcomes related to FDG‐PET‐CT response in patients with Hodgkin lymphoma treated with brentuximab‐vedotin at relapse or consolidation. Clin Lymphoma Myeloma Leuk. 2021;21(12):e929‐e937. 34366266 10.1016/j.clml.2021.07.006

